# Spike protein D614G and RdRp P323L: the SARS-CoV-2 mutations associated with severity of COVID-19

**DOI:** 10.5808/GI.2020.18.4.e44

**Published:** 2020-12-07

**Authors:** Subrata K. Biswas, Sonchita R. Mudi

**Affiliations:** 1 Department of Biochemistry and Molecular Biology, Bangabandhu Sheikh Mujib Medical University (BSMMU), Dhaka 1000, Bangladesh; 2 Department of Biochemistry, Kumudini Women’s Medical College, Mirzapur 1940, Bangladesh

**Keywords:** COVID-19, genome sequence, mutation, RNA-dependent RNA polymerase, SARS-CoV-2, spike protein

## Abstract

The severity of coronavirus disease 2019 (COVID-19), caused by the severe acute respiratory syndrome coronavirus 2 (SARS-CoV-2), greatly varies from patient to patient. In the present study, we explored and compared mutation profiles of SARS-CoV-2 isolated from mildly affected and severely affected COVID-19 patients in order to explore any relationship between mutation profile and disease severity. Genomic sequences of SARS-CoV-2 were downloaded from Global Initiative on Sharing Avian Influenza Data (GISAID) database. With the help of Genome Detective Coronavirus Typing Tool, genomic sequences were aligned with the Wuhan seafood market pneumonia virus reference sequence and all the mutations were identified. Distribution of mutant variants was then compared between mildly and severely affected groups. Among the numerous mutations detected, 14408C>T and 23403A>G mutations resulting in RNA-dependent RNA polymerase (RdRp) P323L and spike protein D614G mutations, respectively, were found predominantly in severely affected group (>82%) compared with mildly affected group (<46%, p < 0.001). The 241C>T mutation in the non-coding region of the genome was also found predominantly in severely affected group (p < 0.001). The 3037C>T, a silent mutation, also appeared in relatively high frequency in severely affected group compared with mildly affected group, but the difference was not statistically significant (p = 0.06). We concluded that spike protein D614G and RdRp P323L mutations in SARS-CoV-2 are associated with severity of COVID-19. Further studies will be required to explore whether these mutations have any impact on the severity of disease.

## Introduction

The severe acute respiratory syndrome coronavirus 2 (SARS-CoV-2), the viral pathogen that causes coronavirus disease 2019 (COVID-19), has infected millions of people worldwide just in 8 months [1]. Although majority of the SARS-CoV-2 infected individuals recover after developing mild to moderate symptoms, more than 800,000 people have already been died due to severe form of COVID-19 [1]. Severity of COVID-19 has been found to greatly vary from patient to patient, but it is so far not entirely clear what is responsible for the variable severity of COVID-19 in the population [1,2]. We recently hypothesized that genetic variation in SARS-CoV-2 may explain the variable severity of COVID-19 [2]. To the best of our knowledge, it has not yet been investigated whether mutation profile of SARS-CoV-2 has any relationship with the severity of COVID-19. However, some studies suggested that D614G mutation in the spike protein may contribute to increased infectivity or transmissibility of SARS-CoV-2 leading to increased severity of COVID-19 [3,4]. In fact, spike protein mediates viral entry into the host cell through binding of the virus with host cell receptor angiotensin-converting enzyme-2 (ACE2) [5]. The D614G mutation of the spike protein was observed sometimes in late January 2020 both in Europe and in China, but then this mutation spread first in the Europe and gradually globally [3,4]. Thus the distribution of spike protein D614G mutation has temporal and geographical variation. The RNA-dependent RNA polymerase (RdRp) enzyme of SARS-CoV-2 is the product of RdRp gene that catalyzes replication of viral RNA. Mutation in the RdRp gene was previously found to be associated with overall increase in mutation rate in the viral genome [6]. In the present study, we explored and compared mutation profiles of SARS-CoV-2 isolated from mildly affected and severely affected COVID-19 patients in order to explore relationship between mutation profile and disease severity.

## Methods

**Fig. 1.**
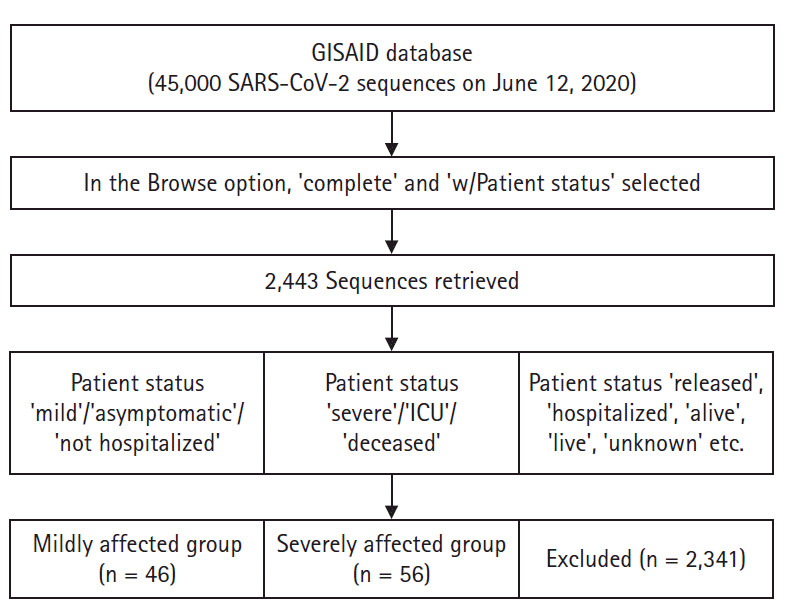
Flow diagram showing sampling procedure. GISAID, Global Initiative on Sharing Avian Influenza Data; ICU, intensive care unit; SARS-CoV-2, severe acute respiratory syndrome coronavirus 2.

Genomic sequences of SARS-CoV-2 were downloaded from Global Initiative on Sharing Avian Influenza Data (GISAID) database (https://www.gisaid.org). In the Browse option, we selected ‘complete’ and ‘w/Patient status’ to retrieve sequences that were complete (>29,000 bases in length) and had patient status information available. Then we explored ‘sample information’ of retrieved sequences, and included sequences in the mildly affected group if patient status was ‘mild’/‘asymptomatic’/‘not hospitalized’. On the other hand, we included sequences in the severely affected group if patient status was ‘severe’/‘ICU’/‘deceased’. Sequences with patient status described with ambiguous words like ‘released’, ‘hospitalized’, ‘alive’, ‘live’, ‘unknown’, etc. were excluded due to uncertainty whether the patients were mildly or severely affected. This sampling procedure is presented as a flow diagram in Fig. 1. Although there were 45,000 SARS-CoV-2 genomic sequences deposited in the GISAID website by June 12, 2020, only 2,443 complete sequences had patient status available. Following the above mentioned search and inclusion/exclusion criteria, we were able to include 46 sequences in the mildly affected group and 56 in the severely affected group (n = 102) in the present study.

Mutation profile was determined using the Genome Detective Coronavirus Typing Tool (available at https://www.genomedetective.com/app/typingtool/cov), a web-based bioinformatics pipeline that can accurately identify changes at nucleotides, coding regions and proteins using a novel dynamic aligner to allow tracking new viral mutations [7]. With the help of this coronavirus typing tool, each of the 102 SARS-CoV-2 sequences was aligned with the Wuhan seafood market pneumonia virus reference sequence NC_045512.3, and all the nucleotide and amino acid sequence variations were identified comparing with the reference sequence. Each of the mutations was counted for mildly and severely affected groups, and expressed in number and percentage. Distribution of selected mutant variants was compared between the mildly affected and severely affected groups by chi-squared test using SPSS version 21.0 (IBM Corp., Armonk, NY, USA). A p-value of less than 0.01 was considered statistically significant.

### Data availability

The mutation profiles of the genomic sequences that support the findings of this study are available from the corresponding author upon reasonable request.

## Results

The SARS-CoV-2 genomic sequences that we included in the present study were sequenced from viral isolates collected in the USA (number; mild, severe: 3, 3), Mexico (0, 3), Brazil (4, 1), Austria (0, 15), Russia (0, 13), Belgium (5, 8), Hungary (1, 0), Spain (1, 3), Turkey (1, 0), Bosnia and Herzegovina (0, 1), India (21, 6), Sri Lanka (0, 1), Japan (3, 0), Indonesia (0, 1), Lebanon (0, 1), Kuwait (1, 0), and Nigeria (6, 0). The viral isolates were collected between February 3 and May 27, 2020. There were 29 men, 13 women and 4 with gender information unavailable in the mildly affected group (n = 46), and 31 men and 25 women in the severely affected group (n=56). Age distribution was 17 to 98 years for mildly affected group and 17 to 93 years for severely affected group.

A comprehensive list of all mutations compared to the Wuhan reference sequence NC_045512.3 in mildly affected and severely affected groups is presented in Supplementary Table 1. In the coding region, there were 103 mutations in the mildly affected group with 37 silent and 66 missense mutations. In the severely affected group, there were 111 mutations with 40 silent and 71 missense mutations. In the non-coding region, there were 2 and 8 mutations in the mildly affected and severely affected groups, respectively, in the 5′ untranslated region (UTR); whereas, there were 9 and 15 mutations in the 3′ UTR in the mildly affected and severely affected groups, respectively. However, majority of the mutations appeared in low frequency, i.e., the mutations were found only in a few cases of mildly and severely affected groups (Supplementary Table 1), and therefore, those mutations were unlikely to be related to the severity of COVID-19.

**Fig. 2.**
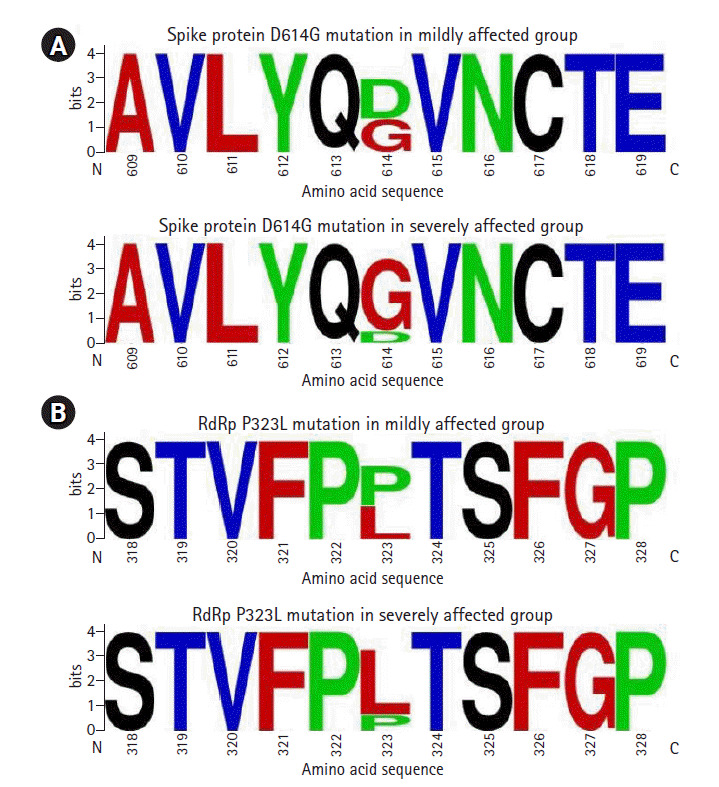
Sequence logos for spike protein (A) around amino acid position 614 and RNA-dependent RNA polymerase (RdRP) (B) around amino acid position 323. Upper panels are for mildly affected group and lower panels are for severely affected group. Selected amino acid sequence is shown on X-axis. The height of each letter on Y-axis indicates its relative frequency. RdRp, RNA-dependent RNA polymerase; N, N-terminal end; C, C-terminal end.

Any mutation with a frequency of 5 or more in either mildly affected or severely affected group is presented in Table 1. In the open reading frame (ORF) 1ab of the SARS-CoV-2 genome, the most frequent mutation identified was 14,408C>T at the nucleotide level. This mutation results in a missense mutation P4715L at the amino acid level of ORF1ab polyprotein that ultimately appears as P323L mutation in the RdRp enzyme. This mutation was predominantly occurred in severely affected group (82.1%) compared with mildly affected group (45.7%, p < 0.001) (Table 1, Fig. 2). In ORF1ab, 11083G>T mutation at the nucleotide level caused L3606F mutation at the amino acid level and involved non-structural protein (nsp) 6. This mutation was found mainly in the mildly affected group (28.3%) compared with severely affected group (1.8%, p < 0.001). Another mutation 5700C>A in ORF1ab caused missense mutation A1812D at the amino acid level. This mutation involved nsp3 and was found in 15.2% of mildly affected group but not found in severely affected group. Among the silent mutations present in ORF1ab, 3037C>T mutation was found more commonly in severely affected group (64.3%) compared with mildly affected group (45.7%, p = 0.06). Other silent mutations in ORF1ab appeared in relatively low frequency in mildly and severely affected groups (Table 1).

In the spike protein, 23403A>G mutation at the nucleotide level resulted in D614G mutation at the amino acid level, and it was predominantly found in severely affected group (85.7%) compared with mildly affected group (45.7%, p < 0.001) (Table 1, Fig. 2). In ORF3a, 25563G>T mutation at the nucleotide level resulted in Q57H mutation at the amino acid level, and this mutation was more prevalent in severely affected group (26.8%) compared with mildly affected group (10.9%, p = 0.08). In ORF8, 28144T>C mutation at the nucleotide level resulted in L84S mutation at the amino acid level. This mutation was found in 26.1% of mildly affected group and in 5.4% of severely affected group (p = 0.008). Other mutations affecting spike, membrane and nucleocapsid proteins of SARS-CoV-2 genome appeared in low frequency (Table 1).

Among all the mutations in the non-coding region of SARS-CoV-2 genome, the 241C>T mutation in the 5´ UTR appeared most predominantly in severely affected group (85.7%) compared with mildly affected group (45.7%, p < 0.001) (Table 1). The 29742G>A, 29827A>T and 29830G>T mutations in the 3′ UTR appeared at a frequency of 17.4%, 34.8%, and 43.5%, respectively, in mildly affected group; but none of these mutations was found in the severely affected group (Table 1).

Of note, in the mildly affected group, four most common mutations (241C>T, 3037C>T, 14408C>T, and 23403A>G) coincided. In the severely affected group, however, 241C>T, and 23403A>G coincided, and 3037C>T and 14408C>T occurred in subsets of them. There was temporal and geographical variation in the distribution of 23403A>G mutation that cause D614G mutation in the spike protein of SARS-CoV-2 [3,4]. In Table 2, we showed collection period of viral isolates in month and frequency of D614G mutation in mildly affected and severely affected groups. Although the percentage of D614G mutation gradually increased from February towards May in both groups, there was more D614G mutation in severely affected group compared with mildly affected group in March (68.2% vs. 45.8%) and April (96.4% vs. 60.0%) when most of the viral isolates were collected for sequencing (Table 2). This finding suggests that increased spike protein D614G mutation in severely affected group was unlikely to be due to temporal variation in the distribution of the mutation. However, in this study, majority of samples of mildly affected group were from India whereas those of severely affected group were from Europe, as described above. To explore whether this fact contributed to the increased spike protein D614G mutation in severely affected group, we showed frequency of D614G mutation in mildly affected and severely affected groups for India and Belgium in Table 3. Of note, the percentage of D614G mutation was found higher in severely affected group compared with mildly affected group for both India and Belgium (Table 3). But such comparisons were not possible for other countries included in this study because very small number of samples was found in either mildly affected or severely affected group for countries other than India and Belgium, as described above.

## Discussion

The severity of COVID-19 greatly varies from patient to patient. Majority of the patients either remain asymptomatic or develop mild to moderate symptoms. However, some COVID-19 patients who develop severe disease die even after hospitalization and intensive care [1]. Why the disease severity differs so much from one person to another is one of the mysteries scientists are still trying to solve [1]. The present study was designed to explore whether genetic variation in SARS-CoV-2 can explain variable severity of COVID-19. Mutation profiles of SARS-CoV-2 isolated from mildly affected and severely affected COVID-19 patients were explored and compared. Among numerous mutations observed in this study, two missense mutations, 14408C>T and 23403A>G, affecting RdRp and spike protein genes, respectively, were found most predominantly in the severely affected group compared with mildly affected group. Along with these two mutations, 241C>T in the 5′ UTR and a silent mutation 3037C>T in the ORF1ab were predominantly found in severely affected group (the later not significantly), however, these mutations do not alter amino acid sequence in a protein. Many other mutations that were found in low frequency in the present study are unlikely to exert an effect on the severity of COVID-19. Thus the ability of spike protein and RdRp mutations on the severity of COVID-19 needs to be considered.

The spike protein of SARS-CoV-2 is responsible for binding with host cell receptor ACE2, and thus it allows entry of the virus into the host cell [5]. In fact, the spike protein of SARS-CoV-2 has 10 to 20 folds higher affinity for ACE2 receptor than the corresponding spike protein of SARS-CoV [8]. Thus the spike protein is potentially related to the infectivity of SARS-CoV-2. The 23403A>G mutation in the genome of SARS-CoV-2 causes replacement of aspartic acid (D) with glycine (G) at position 614 (D614G) of the spike protein. This D614G spike protein mutation appeared sometimes in late January 2020 and then it has spread initially in Europe and then all over the world [3,4]. Several ways have been proposed through which spike protein D614G mutation may increase the infectivity of SARS-CoV-2 [3]. However, computer-based structural analysis of spike protein with D614G mutation suggested that the mutation is unlikely to alter its interaction with human ACE2 receptor [4]. But Korber et al. [3] found that patients infected with spike protein D614G mutant form of SARS-CoV-2 had higher viral loads since fewer PCR cycles were needed for their diagnosis. Furthermore, in cell culture experiment, viral particles with spike protein D614G mutation was found to infect ACE2 expressing cells more efficiently, and this increased infectivity was found to correlate with less shedding of S1 domain of spike protein and more incorporation of spike protein in the virion [9]. In spite of these facts, previous studies were unable to explore an association between the spike protein D614G mutation and disease severity due to relative lack of clinical data of the patients included in their studies [3,4].

As we found in the present study, previous studies also identified that the spike protein D614G mutation frequently accompanies a silent mutation 3037C>T and a missense mutation 14,408C>T in ORF1ab [3]. The 14408C>T mutation in ORF1ab replaces a proline (P) with leucine (L) at position 4715 (P4715L) of ORF1ab polyprotein which actually appears as a replacement of proline with leucine at position 323 (P323L) of RdRp enzyme. The RdRp enzyme of SARS-CoV-2 catalyzes replication of viral RNA and it possesses proof-reading capability [6]. Thus a critical mutation in RdRp gene has the potential to alter viral replication capability with fidelity, and thereby a mutation in RdRp may contribute to infectivity of the virus and severity of the disease. The presence of 14408C>T mutation in SARS-CoV-2 genome that causes RdRp P323L mutation was found to be associated with overall increase in mutation rate in the viral genome [6]. Although the 14408C>T (RdRp P323L) mutation was predominantly found in severely affected patients in the present study, further studies will be required to elucidate whether this RdRp mutation has any significant impact on the viability and infectivity of SARS-CoV-2 and the severity of COVID-19.

The vast majority of genomic sequences of SARS-CoV-2 available at GISAID database do not contain patient status information. Even many of the sequences that contain patient status information use such ambiguous words to describe the information that do not reflect the severity status of the patient. That’s why we were unable to include large number of sequences to analyze in the present study. For the same reason, we were unable to include sequences in such a way that biases due to geographical, temporal, ethnic and gender variations could have been avoided. In spite of small sample size, month wise and country wise analyses of our data suggested that the temporal and geographical variation in the distribution of mutation did not influence our findings to a large extent. However, we do not know whether the patients included in the mildly affected group subsequently developed severe disease or not. In fact, GISAID database does not include any follow up information about patient status. Thus, it is another limitation of the present study, and to overcome this limitation, further studies will be required in which follow up information on patient status is available.

In spite of all these limitations, in the present study, to the best our knowledge, we for the first time compared mutation profiles of SARS-CoV-2 between mildly affected and severely affected COVID-19 patients. Based on our findings, it may be concluded that the spike protein D614G and RdRp P323L mutations predominate in severely affected COVID-19 patients. Further studies will be required to explore whether spike protein D614G mutation or RdRp P323L mutation or the combination of both mutations can exert an impact on the severity of COVID-19.

## Figures and Tables

**Table 1.** Selected mutations in SARS-CoV-2 sequence in mildly and severely affected COVID-19 patients

Gene/genomic region	Nucleotide variation	Amino acid variation	No. of mutations (%)	
			Mild (n = 46)	Severe (n = 56)
5′ UTR	241C>T	N/A	21 (45.7)	48 (85.7)
ORF1ab	313C>T	---	6 (13.0)	0
	3037C>T	---	21 (45.7)	36 (64.3)
	5700C>A	A1812D (nsp3)	7 (15.2)	0
	8782C>T	---	12 (26.1)	3 (5.4)
	11083G>T	L3606F (nsp6)	13 (28.3)	1 (1.8)
	14408C>T	P4715L (RdRp)	21 (45.7)	46 (82.1)
	14805C>T	---	5 (10.9)	2 (3.6)
	15324C>T	---	1 (2.2)	5 (8.9)
	15957G>T	---	0	5 (8.9)
	18877C>T	---	1 (2.2)	11 (19.6)
	20268A>G	---	3 (6.5)	8 (14.3)
S (spike)	22468G>T	---	8 (17.4)	0
	23403A>G	D614G	21 (45.7)	48 (85.7)
	24197G>T	A879S	0	5 (8.9)
ORF3a	25563G>T	Q57H	5 (10.9)	15 (26.8)
M (membrane)	26735C>T	---	0	9 (16.1)
ORF8	28144T>C	L84S	12 (26.1)	3 (5.4)
N (nucleocapsid)	28854C>T	S194L	0	5 (8.9)
	28878G>A	S202N	8 (17.4)	0
	28881G>A, 28882G>A	R203K	9 (19.6)	14 (25.0)
	28883G>C	G204R	9 (19.6)	13 (23.2)
3′ UTR	29742G>A	N/A	8 (17.4)	0
	29827A>T	N/A	16 (34.8)	0
	29830G>T	N/A	20 (43.5)	0

Any mutation with a frequency of 5 or more in either mildly affected or severely affected group is included in this Table. Positions of nucleotides are numbered continuously irrespective of gene or genomic region, and positions of amino acids are numbered separately for each protein. SARS-CoV-2, severe acute respiratory syndrome coronavirus 2; COVID-19, coronavirus disease 2019; Mild, mildly affected group; Severe, severely affected group; UTR, untranslated region; ORF, open reading frame; N/A, not applicable; ---, silent mutation; nsp, non-structural protein; RdRp, RNA-dependent RNA polymerase.

**Table 2.** Distribution of spike protein D614G mutation with collection time in mildly and severely affected COVID-19 patients

Month	Mildly affected group		Severely affected group	
	No. of samples	No. of D614G mutations (%)	No. of samples	No. of D614G mutations (%)
March	24	11 (45.8)	22	15 (68.2)
April	15	9 (60.0)	28	27 (96.4)
May	0	0	6	6 (100)
Total	46	21 (45.7)	56	48 (85.7)

COVID-19, coronavirus disease 2019.

**Table 3.** Frequency of spike protein D614G mutation in mildly and severely affected groups for India and Belgium

Country	Mildly affected group		Severely affected group	
	No. of samples	No. of D614G mutation (%)	No. of samples	No. of D614G mutation (%)
India	21	9 (42.8)	6	6 (100)
Belgium	5	3 (60.0)	8	8 (100)

## Supplementary Materials

Supplementary Table 1. All mutations in the SARS-CoV-2 genome from mildly and severely affected COVID-19 patients
