# Supplementary material for: Spike protein D614G and RdRp P323L: the SARS-CoV-2 mutations associated with severity of COVID-19
Source: Genomics Inform. 2020 Dec 7;18(4):e44. doi: 10.5808/GI.2020.18.4.e44 (PMC7808873; doi:10.5808/GI.2020.18.4.e44)
Supplement: Supplementary Table 1. — All mutations in the SARS-CoV-2 genome from mildly and severely affected COVID-19 patients [file gi-2020-18-4-e44-suppl.pdf]

Supplementary Table 1. All mutations in the SARS-CoV-2 genome from mildly and severely affected COVID-19 patients

Gene/genomic region	Nucleotide variation	Amino acid variation	No. of mutations	
			Mild (n = 46)	Severe (n = 56)
5′ UTR	22A>T	N/A	0	2
	25T>C	N/A	0	1
	25T>G	N/A	0	1
	40C>A	N/A	0	1
	41T>C	N/A	0	1
	204G>T	N/A	1	0
	219G>T	N/A	0	1
	222C>T	N/A	0	2
	241C>T	N/A	21	48
ORF1ab	313C>T	---	6	0
	490T>A	D75E (Leader prot)	0	1
	710C>T	L149F (Leader prot)	0	1
	884C>T	R207C (nsp2)	1	0
	1059C>T	T265I (nsp2)	4	2
	1348C>T	---	1	0
	1397G>A	V378I (nsp2)	2	0
	1440G>A	G392D (nsp2)	0	1
	1457C>T	R398C (nsp2)	0	1
	1541C>T	R426C (nsp2)	1	0
	1594C>T	---	0	1
	2113C>T	---	1	1
	2306C>T	L681F (nsp2)	0	1
	2388C>T	T708I (nsp2)	1	0
	2480A>G	I739V (nsp2)	1	0
	2550A>G	D762G (nsp2)	3	0
	2558C>T	P765S (nsp2)	2	0
	2836C>T	---	0	3
	2891G>A	A876T (nsp3)	0	1
	3037C>T	---	21	36
	3039A>G	Y925C (nsp3)	1	0
	3177C>T	P971L (nsp3)	0	1
	3270T>C	I1002T (nsp3)	0	1
	3371G>T	D1036Y (nsp3)	1	0
	3455T>C	Y1064H (nsp3)	0	1
	3602C>T	H1113Y (nsp3)	0	1
	3634C>T	---	1	2
	3874C>T	---	1	0
	4002C>T	T1246I (nsp3)	0	2
	4011T>C	L1249P (nsp3)	0	1
	4582C>T	---	0	1
	4679C>T	P1472S (nsp3)	3	0
	5230G>T	K1655N (nsp3)	1	0
	5700C>A	A1812D (nsp3)	7	0
	5784C>T	T1840I (nsp3)	1	0
	6040C>T	---	0	1
	6190C>T	---	1	0
	6310C>A	S2015R (nsp3)	2	0
	6312C>A	T2016K (nsp3)	4	0

6507A>G	N2081S (nsp3)	1	0
7164C>T	T2300I (nsp3)	1	0
7765C>T	---	1	1
7823C>T	H2520Y (nsp3)	1	0
8653G>T	M2796I (nsp4)	1	0
8782C>T	---	12	3
8950C>T	---	0	1
9166C>T	---	1	0
9477T>A	F3071Y (nsp4)	1	0
9628T>C	---	1	0
9656A>G	M3131V (nsp4)	1	0
9880T>C	---	3	0
9993A>T	N3243I (nsp4)	0	1
10097G>A	G3278S (3C-like proteinase)	0	2
10771T>C	---	1	0
11083G>T	L3606F (nsp6)	13	1
11093G>A	A3610T (nsp6)	0	1
11109C>T	A3615V (nsp6)	0	1
11398T>C	---	1	0
11457T>C	I3731T (nsp6)	0	2
11518C>T	---	1	0
11563C>T	---	1	0
11822A>G	I3853V (nsp6)	0	1
11842G>T	Q3859H (nsp6)	0	1
12049C>T	---	0	1
12312C>T	A4016V (nsp8)	1	0
12439C>A	---	1	0
12478G>A	M4071I (nsp8)	0	1
12521G>A	G4086S (nsp8)	1	0
12541A>G	---	1	0
12781C>T	---	1	0
13536C>T	---	0	2
13542T>C	---	1	0
13639G>A	D4459N (RdRp)	1	0
13730C>T	A4489V (RdRp)	4	0
13860C>T	---	0	1
14195C>A	T4644N (RdRp)	0	1
14230C>T	P4656S (RdRp)	1	0
14407C>T	P4715S (RdRp)	0	1
14408C>T	P4715L (RdRp)	21	46
14636C>T	A4791V (RdRp)	0	1
14805C>T	---	5	2
15246A>G	---	0	1
15324C>T	---	1	5
15380G>T	S5039I (RdRp)	1	0
15603C>T	---	1	0
15857C>T	T5198I (RdRp)	3	0
15957G>T	---	0	5
16078G>A	V5272I (RdRp)	0	1
16166A>G	N5301S (RdRp)	0	1
17247T>C	---	2	2
17440C>T	P5726S (helicase)	3	0
17690C>T	S5809L (helicase)	1	1
17694T>C	---	0	1

	17747C>T	P5828L (helicase)	2	2
	17808G>T	K5848N (helicase)	1	0
	17858A>G	Y5865C (helicase)	2	2
	18052A>G	T5930A (3-to-5 exonuclease)	1	0
	18060C>T	---	2	2
	18457C>T	P6065S (3-to-5 exonuclease)	0	2
	18736T>C	F6158L (3-to-5 exonuclease)	0	1
	18877C>T	---	1	11
	19101G>T	Q354H (3-to-5 exonuclease)	0	1
	19524C>T	---	2	0
	19816G>T	V6518L (endoRNAse)	1	0
	20081C>T	S6606F (endoRNAse)	3	0
	20134G>T	V6624L (endoRNAse)	1	0
	20268A>G	---	3	8
	20719C>T	---	0	1
	20773G>T	G6837C (2-O-ribose methyl..)	1	0
	21452G>T	G7063V (2-O-ribose methyl..)	1	0
S (spike)	21627C>T	T22I	1	0
	21707C>T	H49Y	0	1
	21805C>T	---	0	1
	21976T>C	---	0	1
	22081G>T	Q173H	0	1
	22155A>G	D198G	0	2
	22444C>T	---	0	4
	22450C>T	---	0	1
	22468G>T	---	8	0
	22521T>G	V320G	0	1
	22991A>G	S477G	0	1
	23282G>T	D574Y	0	1
	23311G>T	E583D	0	1
	23403A>G	D614G	21	48
	23422C>T	---	0	1
	23426G>T	V622F	0	1
	23593G>T	Q677H	1	0
	23731C>T	---	0	2
	23815T>C	---	3	0
	23929C>T	---	4	0
	23952T>G	F797C	1	0
	24034C>T	---	0	1
	24040G>T	---	0	1
	24197G>T	A879S	0	5
	24501T>C	I980T	0	1
	24621C>T	A1020V	0	1
	24642C>T	T1027I	0	1
	24694A>T	---	0	1
	25019G>T	D1153Y	0	1
	25098T>A	I1179N	1	0
ORF3a	25483G>A	A31T	1	0

	25563G>T	Q57H	5	15
	25596A>G	---	1	0
	25721C>T	A110V	1	0
	25979G>T	G196V	1	0
	26144G>T	G251V	4	2
E (envelop)	26265A>T	E7D	0	1
	26338G>T	A32X	1	0
	26351_26356delCGCTTC	A36G, L37_R38del	3	0
M (membrane)	26530A>G	D3G	0	2
	26607C>T	L29F	0	1
	26642C>T	---	1	0
	26729T>C	---	0	1
	26735C>T	---	0	9
	26750C>T	---	0	2
	26779G>T	C86F	1	0
	27046C>T	T175M	1	2
	27093T>A	S191T	0	1
ORF7a	27415G>T	A8S	0	1
ORF7b	27770A>T	---	0	1
	27855C>T	---	0	1
	27861G>T	D36Y	0	1
ORF8	27964C>T	S24L	2	0
	28045C>T	A51V	0	1
	28077G>C	V62L	0	1
	28144T>C	L84S	12	3
	28194C>T	R101C	0	1
	28253C>T	---	1	0
N (nucleocapsid)	28311C>T	P13L	4	0
	28329G>A	G19E	0	1
	28362G>T	G30V	1	0
	28395G>C	R41P	0	1
	28512C>T	P80L	1	0
	28657C>T	---	1	0
	28688T>C	---	2	0
	28690G>T	L139F	0	1
	28765A>G	---	0	1
	28795A>G	---	1	0
	28854C>T	S194L	0	5
	28863C>T	S197L	1	0
	28878G>A	S202N	8	0
	28881G>A, 28882G>A	R203K	9	14
	28883G>C	G204R	9	13
	28896C>G	A208G	0	1
	29236C>T	---	0	2
	29291G>A	D340N	0	1
	29315G>C	D348H	0	1
	29421C>T	P383L	1	0
	29451C>T	T393I	0	1
ORF10	29647A>G	---	0	1
3′ UTR	29700A>G	N/A	0	1
	29705G>T	N/A	3	0
	29711G>T	N/A	0	1
	29734G>C	N/A	0	3

29737G>T	N/A	0	1
29742G>A	N/A	8	0
29742G>T	N/A	2	0
29743C>T	N/A	0	1
29747G>C	N/A	0	1
29759G>T	N/A	0	1
29821T>C	N/A	1	0
29827A>T	N/A	16	0
29829T>A	N/A	1	0
29830G>T	N/A	20	0
29848T>A	N/A	0	1
29850A>T	N/A	0	1
29857C>T	N/A	0	1
29858T>C	N/A	0	1
29862G>A	N/A	0	1
29862G>C	N/A	0	1
29863A>T	N/A	1	0
29867T>A	N/A	1	0
29868G>C	N/A	0	1
29871A>G	N/A	0	1

All mutations in mildly affected and severely affected groups are included in this Table. Positions of nucleotides are numbered continuously irrespective of gene or genomic region, and positions of amino acids are numbered separately for each protein.

SARS-CoV-2, severe acute respiratory syndrome coronavirus 2; COVID-19, coronavirus disease 2019; UTR, untranslated region; N/A, not applicable; ORF, open reading frame; nsp, non-structural protein; RdRp, RNA-dependent RNA polymerase; ---, silent mutation; Mild, mildly affected group; Severe, severely affected group.
